# *HHIP* overexpression inhibits the proliferation, migration and invasion of non-small cell lung cancer

**DOI:** 10.1371/journal.pone.0225755

**Published:** 2019-11-25

**Authors:** Jian-Guo Zhao, Jian-Fang Wang, Jiang-Feng Feng, Xue-Ying Jin, Wan-Li Ye

**Affiliations:** 1 Department of Oncology, Shaoxing People's Hospital (Shaoxing Hospital, Zhejiang University School of Medicine), Shaoxing, Zhejiang, China; 2 Department of Radiotherapy, Shaoxing People's Hospital (Shaoxing Hospital, Zhejiang University School of Medicine), Shaoxing, Zhejiang, China; Chinese Academy of Sciences, CHINA

## Abstract

**Objective:**

The primary purpose of this study is to investigate the effect of hedgehog-interacting protein (*HHIP*) overexpression on the proliferation, migration and invasion of non-small cell lung cancer (NSCLC).

**Methods:**

Firstly, *HHIP* gene expression data of NSCLC tissues and normal tissues were obtained from GSE18842/GSE19804/GSE43458 databases of the Gene Expression Omnibus (GEO) database and then validated by TCGA NSCLC database in a cohort of 1027 cases of NSCLC patients and 108 cases of normal people. A chi-square test was used to analyze the relationship between *HHIP* expression and clinicopathological characteristics of NSCLC. The expression levels of *HHIP* in NSCLC cells were detected by quantitative-real time PCR. The function of *HHIP* was investigated by a series of *in vitro* assays. CCK-8, wounding healing, Transwell invasion assay were utilized to explore the mechanisms of *HHIP*.

**Results:**

*HHIP* mRNA were significantly down-regulated in NSCLC in three GEO databases and TCGA database (P<0.05). This result was confirmed in NSCLC cell lines by qRT-PCR analysis, its expression in normal NSCLC cell line BEAS-2B was significantly higher than that in NSCLC cells. Chi-square test results showed that the low expression of *HHIP* was correlated with gender, cancer type, TNM stage and tumor size. Functional experimental results showed that over-expressing *HHIP* significantly decreased the ability of cell proliferation, migration and invasion in NSCLC cells (P<0.05).

**Conclusion:**

Overall, the above results indicated that *HHIP* could regulate proliferation, migration and invasion, and could be used as a judging criterion for identifying NSCLC classification and stage.

## Introduction

Lung cancer is one of the most common malignant tumors in recent years[1]. According to histology and pathology, it can be divided into small cell lung cancer (SCLC) and non-small cell lung cancer (NSCLC). NSCLC is a common type of lung cancer and accounts for about 85% of the total amount. The pathological type of NSCLC includes lung adenocarcinoma, lung squamous cell carcinoma and large cell lung cancer[2]. The latest statistics shows that about 26% tumor patients died from lung cancer[3]. The mortality of lung cancer is still high in our country and its therapeutic efficacy is limited. Therefore, the discussion of the mechanism of the occurrence and development of NSCLC is of great importance, which may provide clinical therapy with new strategies.

Gene of protein interacting with human sonic hedgehog of *HHIP* is located at 4q31.21–31.3[4], which can compete with the PTCH gene for binding to Hedgehog (Hh) protein, thereby blocking HH signaling. *HHIP* gene is a negative feedback factor in this pathway, which can directly inhibit HH pathway and has extremely important anti-tumor significance[5–7]. Studies have shown that *HHIP* mRNA is expressed in normal tissues, but its expression decreased in some tumor tissues. For example, the low expression of *HHIP* in stromal cells promotes the proliferation of leukemia cells; overexpression *HHIP* attenuates the activation of HGF/MET and HH pathways, and significantly inhibits lung adenocarcinoma cell proliferation, colony formation, invasion and sphere formation under the condition of serum starvation[8]. Meanwhile, other studies have shown that inhibiting the methylation of *HHIP* promoter can inhibit the proliferation and migration of human gastric cancer cells[9]. *HHIP* may be used as an inhibitor of cell metastasis and a prognostic biomarker in gastric cancer[10]. However, the relationship between *HHIP* expression and NSCLC has not been elucidated.

## Materials and methods

### Data resource

Data of NSCLC chips, including GSE18842, GSE19804 and GSE43458, were obtained from Gene Expression Omnibus (GEO) (http://www.ncbi.nlm.nih.gov/geo/). GEO query package was used to download the data of *HHIP* expression. Datasets GSE19804 (S1 Table) and GSE18842 (S2 Table) respectively included 60 and 44 paired lung tumors and adjacent non-tumor tissues from patients of NSCLC. Dataset GSE43458 (S3 Table) contained 30 normal control samples and 80 NSCLC samples. Student’s *t* test was conducted to analyzed the differential expression of *HHIP* in lung cancer. Meanwhile, gene expression profiles of NSCLC were obtained from TCGA database, including clinicopathological features like patient’s age at diagnosis, gender, race, diagnostic classification, tumor stage, tumor grade and other basic information (S4 Table). In order to eliminate other interference factors as possible, we only chose samples with complete clinical information (S5 Table) for analysis the significance of *HHIP* expression in NSCLC.

### Experimental materials and reagents

Human normal lung cell line (BEAS-2B) and NSCLC cell lines (H1975, H358, H226 and HCC827) were purchased from ATCC (USA); RPMI-1640 medium, fetal bovine serum (FBS) and 0.25% trypsin were purchased from Gibco (USA); Trizol and Lipofectamine^TM^2000 were purchased from ThermoFisher (USA); pEGFP overexpression plasmid was purchased from Shanghai Beinuo Biotechnology Co., Ltd.; CCK-8 kit was purchased from Shanghai Beyotime Biotechnology Co., Ltd.; cDNA PrimeScript RT kit and SYBR-Green PCR Master Mix kit were purchased from Dalian TaKaRa Co., Ltd.; Transwell chamber was purchased from Milipore (USA); artificial Matrigel was purchased from BD Biosciences (USA). The other reagents were all domestically analytical reagents. ABI 7500 fluorescence quantitative PCR was purchased from Applied Biosystems (USA).

### Cell culture

BEAS-2B, H1975, HCC827, H226 and H385 cell lines were cultured in RPMI-1640 culture solution containing 10% FBS, 100U/mL of penicillin and 100mg/mL of streptomycin. All cells were kept in an incubator with 5% CO_2_ at 37°C. When the cells attached and the density reached 70%-80%, cells were digested with 0.25% pancreatin. Cells in logarithmic growth phase were selected for subsequent experiments.

### Cell transfection

Lung cancer cells in logarithmic growth phase with a density of 3×10^6^ cells/mL were added into a 6-well plate. Then, 100μL of RPMI-1640 medium containing 10% FBS was added to each well respectively. When the cell reached 60%-70% confluent, 1,000μL of FBS-free medium was replaced. Then, 10μL of Lipofectamine^TM^2000 and *HHIP*-pEGFP recombinant plasmid was added to each tube of Lipofectamine^TM^2000 Reagent for incubating 30 min at room temperature. Finally, complex was cultured in a 6-well plate for 6h, then add 1,000μL of FBS. After transfected for 48h and observed with fluorescence microscope for subsequent experiments. The cells were divided into three groups: blank control group; NC-vector group, lung cancer cells transfected with pEGFP vector; *HHIP*-vector group, lung cancer cells transfected with *HHIP*-pEGFP.

### The detection of *HHIP* mRNA expression with real-time PCR

Total RNAs in NSCLC cells were extracted with Trizol according to the manufacturer’s protocol. After determination of RNA concentration and purity, cDNA was synthesized with PrimeScript RT kit and amplified with PCR. The expression of *HHIP* was detected with SYBR-Green PCR Master Mix kit, then the expression of *HHIP* mRNA was detected taking the GAPDH as internal reference. The following primer sequences were used: *HHIP* Forward Primer: 5’- TCTCAAAGCCTGTTCCACTCA -3’, Reverse Primer: 5’-GCCTCGGCAAGTGTAAAAGAA-3’; GAPDH: Forward Primer: 5’- TGACTTCAACAGCGACACCCA -3’, Reverse Primer: 5’- CACCCTGTTGCTGTAGCCAAA -3’

The reaction system of PCR was 10μL and the reaction conditions were as below: 3 min of pre-denaturation at 95°C; 10s for 95°C, 30s for 59°C and 30s for 72°C with a total of 40 cycles. The relative expression of *HHIP* was analyzed by relative quantification method, and data was presented in the form of Folds = 2^-△△Ct^. Primers used in the experiment were designed by Primer3 and synthesized by BGI group.

### The detection of cell proliferation with CCK-8 assay

Cells in logarithmic growth phase of each group were seeded into 96-well plate to culture with a density of 3×10^3^ cells/well, and then transfected with NC, NC-vector and HHIP-vector. Cells were cultured for 0h, 24h, 48h, 72h, 5 repeated holes were set in each group and zero adjustment was performed at blank hole. Then, CCK-8 reagent was added following the instruction and cells were put into the incubator for 2h. Enzyme mark instrument was used to examine the absorbance value of each hole at 450nm. The assay was repeated 3 times and recorded the OD value and drawn the cell growth curve.

### The detection of cell migration ability with Wound Healing assay

To detect the abilities of migration, after transfection for 12h, H1975 and HCC827 cells were inoculated in 6-well plate to culture. When the cell fusion of each group reached 90%, 10μL of pipette tip was used to scratch in the shape of “—”. The cells were washed with PBS 3 times and replaced with fresh medium for allowing to migrate for 48 h. Wound healing rate (%) = (scratch width at 0h - scratch width at 48h)/ scratch width at 0h*100%.

### The detection of cell invasion ability with Transwell assay

Transwell chamber inserts with Matrigel were used for invasion assay. After transfection, cells of each group were resuspended in serum-free culture solution. Then, 100μL of cell suspension of each group were seeded into the upper chamber with a total number of 6×10^4^ cells and 400μL culture solution containing 10% FBS was added into the lower chamber for allowing to invade for 48 h. The non-invasion cells were wiped out with a cotton swab. The cells in the upper chamber were fixed with 10% formaldehyde, stained with crystal violet for 3 min and counted under an inverted microscope of 5 fields.

### Statistical analysis

Experimental data were analyzed by SPSS 19.0 or Graph Pad Prism 7 software, and presented in the form of mean value ± standard deviation (SD). Comparisons among groups were analyzed by paired samples t test or independent samples t test. Research data were all from independent assays that were repeated three times or more. P < 0.05 was considered to be statistically significant.

## Results

### The expression of *HHIP* significantly decreased in samples and cell strains of NSCLC patients

**Fig 1:**
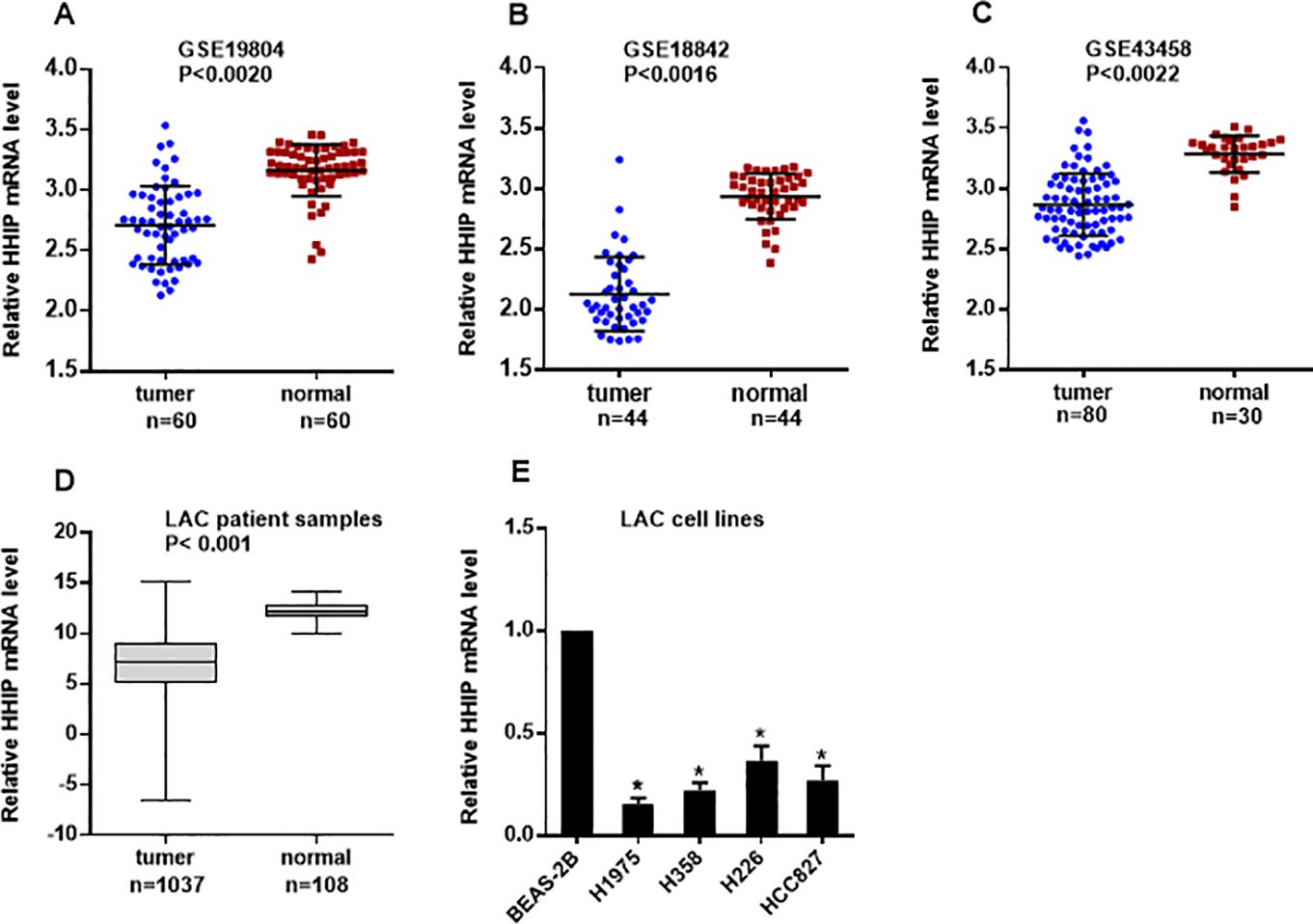
*HHIP* had lower expression in LAC cancer patients and LAC cell lines compared to normal control. (A) Analysis of the GEO dataset GSE19804 indicated that *HHIP* mRNA expression was decreased in lung cancers compared to the normal controls(n = 60,mean±SD, with paired t-test).(B) Analysis of the GEO dataset GSE18842 indicated that *HHIP* mRNA expression was decreased in lung cancers(n = 46) compared with normal lung tissues(n = 45). (mean ± SD, with paired t-test, all samples were paired except 3). (C) Analysis of the GEO dataset GSE 43458 indicated there was decreased HHIP mRNA expression in the 80 lung adenocarcinomas compared to normal lung tissue(n = 30). (mean ± SD, with unpaired t-test). (D). Analysis of the TCGA indicated that *HHIP* mRNA expression was significantly downregulated in the lung cancer samples (n = 1037) compared to the normal controls (n = 108). (mean ± SD, with paired t-test). (E) The relative HHIP expression in different LAC cell lines. The *HHIP* expression was compared to that of non-tumor lung cell line BEAS-2B (which is set as 1).

Firstly, the *HHIP* expression in lung cancer was investigated. Three lung cancer microarray datasets were obtained from GEO database in NCBI. Paired samples t test was performed for 60 cases of lung adenocarcinoma tissues (2.709±0.323) and adjacent normal tissues (3.163±0.21) in GSE19804 and 44 cases of that (2.128±0.308 vs 2.936±0.188) in GSE18842, finding that the expression of *HHIP* mRNA in cancer tissues significantly down-regulated(**Fig 1A** and **1B**) and the difference were statistically significant (P < 0.001). Independent samples *t* test was performed for GSE43458 data, finding that the expression of *HHIP* mRNA of cancer patients (2.865±0.158) was significantly lower than that of normal people (3.285±0.152) (**Fig 1C**).

Then, *HHIP* expression of 1037 cases of NSCLC tissues and 108 cases of normal tissues in TCGA database were obtained. As shown in **Fig 1D**, the expression of *HHIP* in NSCLC tissues (6.981±2.777) significantly decreased compared with normal tissues (12.193±0.860). In order to verify that whether the same result could be concluded in NSCLC cell lines *in vitro*, the expression of *HHIP* in four lung cancer cell lines (H1975, H358, H226, HCC827) and one normal lung cell line (BEAS-2B) were detected. As shown in **Fig 1E**, the expression of *HHIP* in lung cancer cell lines significantly decreased compared with that in BEAS-2B cells. Thus, above results indicated that *HHIP* significantly decreased in NSCLC.

### Analysis of *HHIP* expression-related factors

The 738 cases of NSCLC samples with complete clinical information from TCGA database were divided into two groups to was analyzed the relationship between HHIP expression and clinicopathologic features such as age, gender, metastasis of blood vessel and lymph and clinical stage (TNM), etc. 738 cases of samples were divided into *HHIP* high expression group and *HHIP* low expression group based on the median. Chi-square test showed that *HHIP* expression was not correlated with the age, lymph node metastasis and hematogenous metastasis, but significantly correlated with gender, cancer type, TNM stage and tumor size of patients (*P* < 0.05), as shown in **Table 1**.

**Table 1:** The relationship between *HHIP* expression and clinicopathological features in NSCLC.

Clinicopathological Characteristics	N	*HHIP* High Expression	*HHIP* Low Expression	*x*^*2*^	*P* Value
**Age**					
<60	171	81	90	0.617	0.485
≥60	567	288	279		
**Gender**					
Male	466	206	260	16.978	0.000
Female	272	163	109		
**Cancer Type**					
Adenocarcinoma	337	208	129	34.083	0.000
Carcinoma and others	401	161	240		
**TNM Stage**					
I	365	204	161	10.023	0.002
II-IV	373	165	208		
**Lymph Node Metastasis**					
Yes	272	124	148	3.354	0.079
No	466	245	221		
**Hematogenous Metastasis**					
Yes	28	16	12	0.594	0.564
No	710	353	357		
**Tumor Diameter**					
<3 cm	190	121	69	19.166	0.000
≥3 cm	548	248	300		

### *HHIP* overexpression could inhibit the proliferation of lung cancer cells

**Fig 2:**
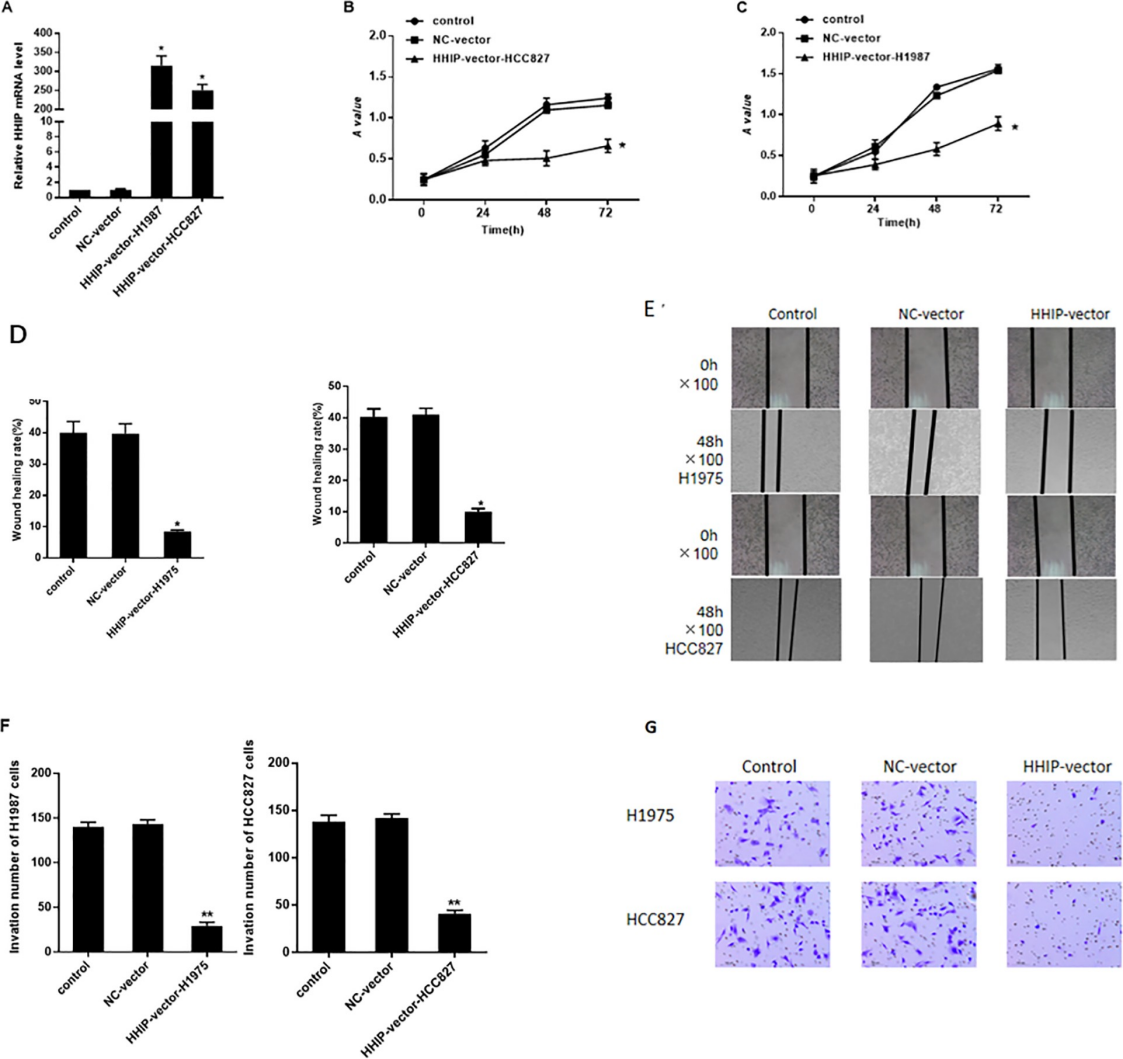
*HHIP* overexpression significantly inhibited cell proliferation, migration and invasion. (A) Relative expression of *HHIP* in H1975 and HCC827 cells linking with p-EGFP vector after RT-PCR detection; (B) Growth curve in vitro of HCC827 cell after over-expressing *HHIP*; (C) Growth curve in vitro of H1975 cell after over-expressing *HHIP*; (D) The detection of the invasion rate of HCC827 and H1975 cells with wound healing assay; (E)Representative pictures at 0h and 48h; (F) The detection of the number of invasive HCC827 and H1975 cells with Transwell assay; (G) Representative pictures. mean value ± SD, with a paired T test; “*” means *P*<0.05, “**” means *P*<0.01.

HCC827 and H1975 cell lines were selected to perform over-expression assay of *HHIP*. As shown in **Fig 2A**, the result of qRT-PCR showed that the expression of *HHIP* mRNA in *HHIP*-vector group significantly increased compared with that in blank control group and negative control group (P < 0.01). As shown in **Fig 2B** and **2C**, the result of CCK-8 assay showed that there was no significant difference on proliferation when cells were treated at 0h, while the proliferation of H1975 and HCC827 cells in HHIP-vector group significantly decreased at 24h, 48h and 72h compared with that in blank control group and negative control group (P < 0.01). Overall, these results suggested that over-expression *HHIP* could inhibit the viability and proliferation of NSCLC cells.

### *HHIP* overexpression could inhibit the migration and invasion of lung cancer cells

Wounding healing and Transwell assays were used to detect cell migration and invasion of lung cancer cells. As shown in **Fig 2D and 2E**, compared with negative control group, there was no statistical difference of the wound healing rate at 48h of blank control group, overexpression of *HHIP* while *HHIP*-vector group significantly decreased (P < 0.01). As shown in **Fig 2F** and **2G**, Transwell assays showed that transfected with *HHIP*-vector significantly decreased the invasion ability of H1975 and HCC827 cells, whereas transfection with NC and NC-vector showed relatively high ability (P < 0.01). The results above indicated that over-expression *HHIP* could significantly inhibit the migration and invasion of H1975 cells.

## Discussion

In recent years, some studies at home and abroad have shown that the over-activation of HH signaling pathway has a certain relationship with tumorigenesis. *HHIP*, acting as a negative regulator of HH signaling pathway, participates in the negative feedback loop of HH signaling pathway regulation. Theoretically, the decreased *HHIP* expression is related to the over-activation of HH signals. In the study of Hedgehog signaling pathway and the expression of various molecules in malignant tumors, the expression of *HHIP* gradually attracted the attention of researchers. Yue *et al*. examined 25 cases of colorectal cancer tissues, they found that *HHIP* was mainly expressed in the crypts of cancer cells in some samples, mainly, and no *HHIP* expression in the interstitial part[11]. Many studies have found that the expression of *HHIP* in malignant tumors is lower than that in normal tissues. However, LingYang *et al*. detected HH signaling protein in 34 cases of ovarian cancer tissues, and found that the expression of *HHIP* increased in 7 cases, which is not related to tumor subtypes, gender and other characteristics[12]. And H Taniguchi *et al*. found that *HHIP* decreased in some cancer tissues of digestive tract tumors and silencing *HHIP* may cause tumor lymph node metastasis and promote tumor development[13].

In this study, we firstly studied the expression of *HHIP* in the GSE datasets and found that the expression of *HHIP* was down-regulated in lung cancer tissues, which was confirmed in the TCGA data that the expression of *HHIP* in 1037 cases of NSCLC tissues was significantly lower than that in normal lung tissue samples (108 cases). Meanwhile, a Chi-square test determined that low *HHIP* expression was significantly correlated with gender, cancer subtype, TNM stage and tumor size. Therefore, *HHIP* might be used as a judging criterion for NSCLC classification and TNM stage. Form subsequent experiments, we found that overexpression of *HHIP* could inhibit the proliferation, migration and invasion of NSCLC cell lines. Li *et al*. found that overexpressing *HHIP* under the condition of serum starvation could significantly inhibit lung adenocarcinoma cell proliferation, colony formation, invasion, and tumor sphere formation, and the significant decreased *HHIP* resulted from epigenetic silencing that was caused by the region methylation of the *HHIP* promoter[8].

The decreased expression of *HHIP* in lung cancer tissues may be caused by various reasons, such as the methylation of CpG island in the promoter region of *HHIP* gene and the loss of heterozygosity (LOH). LOH, a very common DNA mutation in tumor cells, is another mechanism that could inhibit gene expression. Tada *et al*.[14] reported that there was a decreased *HHIP* expression in liver cancer, which was partly due to the methylation of *HHIP* gene promoter, and some were due to LOH. *HHIP* is located on chromosome 4q31.21~31.3 that has been confirmed to be a target for allele loss in malignant tumors such as pancreatic cancer and liver cancer. Therefore, LOH may also occur in *HHIP* gene of NSCLC. In addition to gene methylation and LOH might lead to gene expression silencing, the expression of *HHIP* may also be regulated by microRNAs. MicroRNAs might be involved in tumor development, related to prognosis and therapy as they could regulate cell proliferation, differentiation and apoptosis by regulating the translation process[15].

*HHIP*, a new tumor suppressor, is involved in the occurrence and development of many tumors, but its specific mechanism of its role in each malignant tumor needs further study. *HHIP* may provide a broader clinical perspective to elucidate the occurrence and development of malignant tumors, and the early diagnosis, treatment, and prognosis judgment of malignant tumors.

## Supporting information

S1 Table HHIP expression of NSCLC samples in GEO dataset GSE19804. (XLSX)

S2 Table HHIP expression of NSCLC samples in GEO dataset GSE18842. (XLSX)

S3 Table HHIP expression of NSCLC samples in GEO dataset GSE43458. (XLSX)

S4 Table The clinicopathological features of 1037 NSCLC samples from TCGA database. (XLS)

S5 Table The 738 cases of NSCLC samples with complete clinical information from TCGA database. (XLSX)

S1 Data HHIP expression of NSCLC samples from TCGA database. (TXT)
